# Functional role of autophagy in gastric cancer

**DOI:** 10.18632/oncotarget.7508

**Published:** 2016-02-19

**Authors:** Hao-ran Qian, Yi Yang

**Affiliations:** ^1^ Department of General Surgery, Institute of Micro-Invasive Surgery of Zhejiang University, Sir Run Run Shaw Hospital, Medical College of Zhejiang University, Hangzhou, Zhejiang, PR China; ^2^ Department of Pharmacology, Hangzhou Key Laboratory of Medical Neurobiology, School of Medicine, Hangzhou Normal University, Hangzhou, Zhejiang, PR China

**Keywords:** autophagy, autophagy-related gene, gastric cancer, tumorigenesis, progression

## Abstract

Autophagy is a highly regulated catabolic pathway responsible for the degradation of long-lived proteins and damaged intracellular organelles. Perturbations in autophagy are found in gastric cancer. In host gastric cells, autophagy can be induced by *Helicobacter pylori* (or *H. pylori*) infection, which is associated with the oncogenesis of gastric cancer. In gastric cancer cells, autophagy has both pro-survival and pro-death functions in determining cell fate. Besides, autophagy modulates gastric cancer metastasis by affecting a wide range of pathological events, including extracellular matrix (ECM) degradation, epithelial-to-mesenchymal transition (EMT), tumor angiogenesis, and tumor microenvironment. In addition, some of the autophagy-related proteins, such as Beclin 1, microtubule-associated protein 1 light chain 3 (MAP1-LC3), and p62/sequestosome 1 (SQSTM1) have certain prognostic values for gastric cancer. In this article, we review the recent studies regarding the functional role of autophagy in gastric cancer.

## INTRODUCTION

Gastric cancer is a prevalent digestive tract tumor and one of the leading causes of cancer-related death worldwide. According to the estimates from the International Agency for Research on Cancer (IARC) in 2012, 951,600 cases are newly diagnosed as gastric cancer and this disease contributes to 723,100 deaths [1]. Host-associated factors as well as environmental factors contribute to disease development [2], among which the chronic *Helicobacter pylori* (or *H. pylori*) infection is considered as a major risk factor for tumorigenesis [3]. However, the molecular mechanisms involved in the oncogenesis and progression of gastric cancer have not yet been fully understood.

Autophagy, an intracellular homeostatic pathway, is highly conserved from yeast to human. By degradation of intracellular materials, autophagic process provides cells energy under nutrient-depleted conditions or upon various cellular stresses. According to this view, autophagy serves as a self-defensive pathway which benefits tumor cell survival under extraordinary circumstance by preventing the accumulation of garbage or toxins [4, 5]. However, aberrant autophagic activity may result in the inappropriate degradation of proteins and organelles that are indispensible for maintaining tumor cell survival, and ultimately lead to autophagic cell death [6]. Growing evidence reveals the vital role of autophagy in many physiopathological processes of human diseases, including neurodegenerative diseases [7], vascular disorders [8], inflammation [9], as well as cancer [10].

In this article, we review the recent studies regarding the involvement of autophagy in the tumorigenesis and progression of gastric cancer. The potential clinical significance of autophagy-related proteins in predicting the prognosis of gastric cancer is also discussed. Understanding the functional role of autophagy in modulating gastric cancer pathogenesis allows us to harness this process for improving the disease management.

## AN OVERVIEW OF AUTOPHAGY

**Figure 1:**
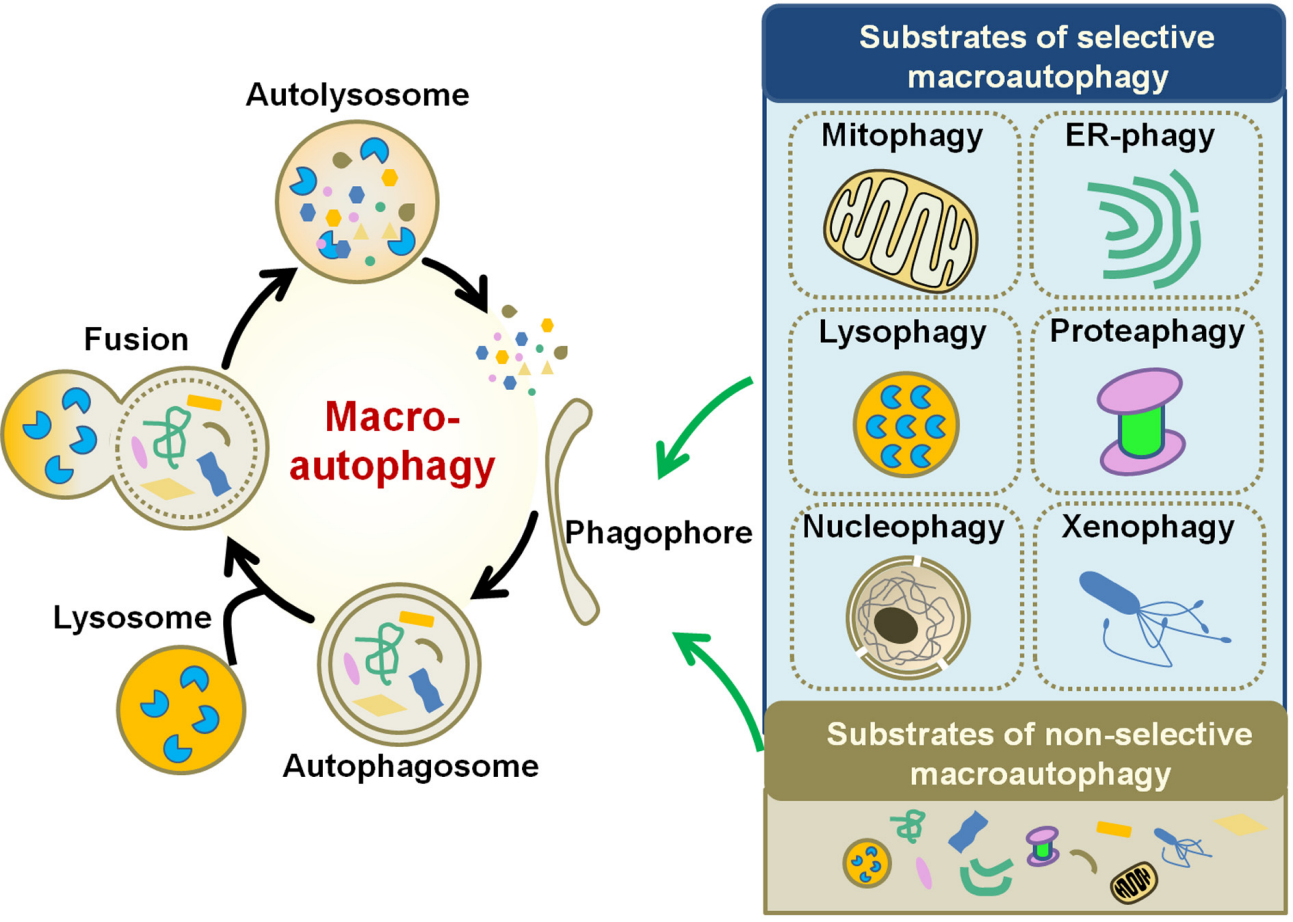
Schematic diagram of macroautophagy Macroautophagy initiates by the formation of phagophore, which sequesters specific cargoes (selective macroautophagy) or bulk cytoplasm (non-selective macroautophagy) into an autophagosome. Fusion of the autophagosome with lysosome results in the degradation of the cargoes. Upon the induction of selective autophagy, the cargoes can be mitochondria (for mitophagy), ER (ER-phagy), lysosomes (lysophagy), proteasomes (proteaphagy), nucleus (nucleophagy), or microbes (xenophagy).

Depending on the choice of path by which cargo is delivered into lysosome, autophagy is categorized into three forms, macroautophagy, microautophagy and chaperone-mediated autophagy (CMA) [11]. Macroautophagy is a process by which the aggregated proteins or damaged intracellular organelles are encapsulated into a double-membrane structure termed autophagosome, which fuses with lysosomes for protein and organelle degradation [12] (Figure 1). Microautophagy is characterized by the direct engulfment of cytoplasmic cargo by the invagination of lysosomal membrane followed with the delivery of the contents into the lysosomal lumen [13]. Both macroautophagy and microautophagy can be selective and non-selective. Non-selective autophagy is responsible for the degradation of the bulk cytoplasm (Figure 1). According to the unique substrate that delivered, selective autophagy is termed as mitophagy for mitochondrial turnover [14], ER-phagy for endoplasmic reticulum (ER) [15, 16], lysophagy for lysosomes [17], proteaphagy for proteasomes [18], nucleophagy for nucleus [16], xenophagy for microbes (bacteria and viruses) [19, 20] (Figure 1). CMA is a selective lysosomal degradative pathway in which cytosolic proteins are delivered to lysosomes for degradation by molecular chaperones [21]. As macroautophagy is accepted as the most universal subtype of autophagy and is known to be involved in the human cancer, in this review we mainly discuss the interaction between macroautophagy (hereafter autophagy) and gastric cancer. The autophagic process involves a series of steps with multiple autophagy-related genes (Atgs) and signaling pathways participated in the core machinery of autophagy [22].

## AUTOPHAGY IN ONCOGENESIS OF GASTRIC CANCER

### Autophagy and *Helicobacter pylori* in gastric cancer

Infection of *H. pylori*, a Gram-negative bacterial pathogen, is considered as a major etiological factor for the gastric carcinogenesis [23]. *H. pylori* virulence factors, such as vacuolating cytotoxin (VacA) and cytotoxin-associated gene A (CagA), contribute to the development of gastric cancer [24]. Emerging lines of evidence suggest that several microorganisms, including *H. pylori*, can be eliminated through an autophagy-dependent degradative pathway known as xenophagy [20]. Moreover, individuals with the *atg16L1* (mammalian Atg16 homologue) risk allele have increased risks to infection [25]. It appears that autophagy is linked to the host susceptibility to *H. pylori* infection.

The association between autophagy and *H. pylori* infection in host cells is multifaceted [26]. Accumulating evidence indicates that *H. pylori* is capable of inducing autophagy in both gastric epithelial cells [27] and professional phagocytes, such as macrophages [28]. In gastric epithelial cells, virulence factor VacA is required for the formation of autophagosomes, and the activation of autophagy in response to VacA exposure in turn limits the toxin-induced host cell damage by degrading VacA [27, 29]. Silencing of the autophagy-related protein Atg12 leads to increased VacA level in infected cells [27]. In agreement with this finding, blockage of autophagy by 3-methyladenine favors the intracellular replication and survival of *H. pylori*, while autophagy stimulator rapamycin promotes bacterial clearance in gastric epithelial cells [30]. These observations point towards the possibility that autophagy may serve as a self-defensive pathway against toxin exposure. In contrast, prolonged exposure of gastric epithelial cells to VacA causes disturbance in autophagy, and consequently results in the excessive production of reactive oxygen species (ROS) and the accumulation of autophagic substrate p62/sequestosome 1 (SQSTM1) [25]. ROS governs the human tumorigenesis through driving DNA mutation [31]. Severe DNA lesion or defective DNA repair capacity, induced by chronic oxidative stress, promotes the disease progression from infection to gastric cancer [32]. The overexpression of the oncoprotein p62/SQSTM1, caused by autophagy defects, has been found to promote tumorigenesis *via* impairing nuclear factor-κB (NF-κB) signaling transduction pathway [33]. In this regard, it is speculated that autophagy in host cells has varied responses to acute and chronic virulence factor exposure.

Unlike VacA, which is necessary and sufficient for the autophagosome formation in gastric epithelial cells, CagA seems to be dispensable for autophagy induction [27]. According to Tsugawa's report [34], VacA activates the autophagic degradation of CagA in host gastric epithelial cells. Interestingly, CagA predominantly accumulates in CD44-positive gastric cancer stem-like cells by escaping from autophagic degradation [34]. The CD44 expression can be induced upon chronic inflammation [35]. Considering the CD44-exresssing cancer stem cells might be developed from normal gastric epithelial cells upon chronic *H. pylori* infection, CagA is more likely to participate in a later stage, rather than early stage of gastric carcinogenesis.

Systemic reviews regarding the *H. pylori*-modulated autophagy in gastric cancer can be found elsewhere [36, 37].

### Atgs in tumorigenesis of gastric cancer

The implication of some autophagy-related proteins in gastric cancer is summarized in Table 1. Beclin 1 (mammalian homologue of yeast Atg6) and microtubule-associated protein 1 light chain 3 (MAP1-LC3 or LC3, mammalian homologue of yeast Atg8) are involved in the biosynthesis of the autophagosome, which is the initial step of autophagy. Increased Beclin 1 [38-40] and LC3-I/II conversion [39] was detected in human gastric cancer tissues. Upregulated Beclin 1 mRNA and protein is also noted in several gastric cancer cell lines, as compared with that in normal gastric mucosa cell line (GES-1) [41, 42]. Using immunohistochemical analysis, the distribution of Beclin 1 is mainly found in cytoplasm of tumor cells. The point mutation of *beclin 1* rarely occurs in common human cancers. According to Lee's report, only 2.8% (5/180) of the gastric cancer samples harbor *beclin 1* gene mutation [43]. Concomitantly, the somatic mutation of *atg5* is presented in gastric cancers although the incidence is quite low (1.5%; 2/135) [44]. It should be noted that the protein expression of Atg5 is lost in 21.0% (21/100) of the gastric cancers [44]. In another study, Vigen et al. reported an extremely high negative expression of Atg5 and Atg16 in patients with gastric adenocarcinoma (Atg5, 80.0%, 8/10; Atg16, 80.0%, 8/10) or gastric carcinoid (Atg5, 60.0%, 6/10; Atg16, 90.0%, 9/10) [40]. Considering the number of samples is relatively small, further integrative work would be required by including considerably larger sample size. Nevertheless, the loss of expression or mutation of these *atgs* may participate in the tumorigenesis and progression of gastric cancer possibly *via* altering the autophagic cell death.

**Table 1:** Autophagy-related proteins and their functions in gastric cancer

Protein	Function in autophagic machinery	Function in gastric cancer
Atg2	Required for autophagosome formation [106]	Frameshift mutations in *atg2B* are common in gastric cancers with high MSI [45]
Atg5	Coupled with Atg12 to form an Atg12-Atg5 conjugate, which is involved in phagophore expansion [107]	Frameshift mutations in *atg5* are common in gastric cancers with high MSI [45]. Absent in approximately one fifth of the gastric cancers [44], but is highly expressed in chemoresistant gastric cancer cells [72]. Associates with the shorter survival time in gastric cancer patients [72]
Atg6/Beclin 1	A component of the class III PI3K complex that contributes to the biosynthesis of phagophore and autophagosomes [107]	Upregulated in gastric cancer tissues [38-40] and several gastric cancer cell lines [41, 42]. Increased Beclin 1 expression predicts a more favorable prognosis [41, 95, 96]
Atg8/LC3	Involved in the cargo recruitment into, and the biosynthesis of autophagosomes [107]	Upregulation of LC3 correlates Ki-67 in gastrointestinal cancers [99]. The number of LC3-positive puncta predicts poor prognosis of gastric cancer [100]
Atg9	Acts as a lipid carrier for phagophore expansion [107]	Frameshift mutations in *atg9B* are common in gastric cancers with high MSI [45]
Atg12	Coupled with Atg5 to form an Atg12-Atg5 conjugate, which is involved in phagophore expansion [107]	Frameshift mutations in *atg12* are common in gastric cancers with high MSI [45]. Contributes to autophagy-mediated degradation of virulence factor VacA in gastric epithelial cells [27]
Atg16	Associates with Atg12-Atg5 conjugate and participates in phagophore expansion [107]	Individuals with the *atg16L1* risk allele have increased risks to *H. pylori* infection [25]. Negatively expressed in gastric cancer [40]
AMPK	An important cellular energy sensor that is able to activate autophagic process [81, 82]	Acts a pro-survival role for cancer cells during ECM detachment by inhibiting mTOR and activating autophagy [81, 83, 84]
Bcl-2	A member of the Bcl-2 family; negatively regulates autophagy by interacting with Beclin 1 [108]	Modulates both autophagy and apoptosis pathways during gastric cancer cell death [75], and is a prognostic factor of survival in gastric cancer patients [93]
mTOR	A protein kinase that negatively modulates macroautophagy [107]	Blockage of mTOR activates autophagy, which subsequently favors cancer cell survival during ECM detachment [83, 84]
p62/SQSTM1	An ubiquitin binding protein that serves as a selective autophagy substrate [116]	Upregulated in gastric cancer; increased p62/SQSTM1 level associates with poor differentiation and reduced lymph node metastasis of gastric cancer [102]
SIRT1	Drives the initiation of autophagy *via* deacetylation of Atgs, such as LC3 [89]	Upregulated in tumor tissues and correlates with advanced lymph node metastasis in gastric cancer [87]. Regulates EMT and invasion capability of gastric cancer cells [88]

AMPK, AMP-activated protein kinase; Atg, autophagy-related gene; Bcl-2, B-cell lymphoma-2; ECM, extracellular matrix; EMT, epithelial-to-mesenchymal transition; LC3, microtubule-associated protein 1 light chain 3; mTOR, mammalian target of rapamycin; MSI, microsatellite instability; PI3K, phosphatidylinositol 3-kinase; SQSTM1, sequestosome 1; SIRT1, silent mating type information regulation 1.

Using single-strand conformation polymorphism analysis, Kang et al. revealed that the frameshift mutations in *atg* genes with mononucleotide repeats, including *atg2B* (mammalian *atg2* homologue), *atg5*, *atg9B* (mammalian *atg9* homologue), and *atg12*, were common in gastric cancers with high microsatellite instability (MSI) subtypes (28.1%; 9/32), while mutations were not detected in patients with low MSI [45]. In addition, the frameshift mutations of ultraviolet radiation resistance-associated gene (UVRAG), which binds to Beclin 1 and triggers autophagy activation, is also found in gastric cancers with high MSI (9.4%; 3/32) [46]. These evidence suggest that the autophagic route might be perturbed especially in gastric cancers with high MSI. Identification of crystal structure of Atg family proteins or complex may provide an essential molecular basis for understanding the unique role of Atgs in the oncogenesis of gastric cancer.

### Pro-death and pro-survival role of autophagy in gastric cancer

Autophagy is considered to play a tumor suppressive function by leading cells to autophagic death [47]. This augment has been supported by numerous evidence [48, 49]. Indeed, autophagic cell death, but not apoptotic cell death, has been documented in gastric cancer death induced by different pharmacological treatments [50, 51]. In contrast, the concurrent induction of autophagy and apoptosis has been documented in gastric cancer cell in a number of studies [52-57]. Although varies largely in molecular mechanisms, the autophagic and apoptotic machinery talk to each other in many aspects [58]. The pattern of crosstalk between autophagy and apoptosis are worthy of consideration here. First, autophagy and apoptosis may govern gastric cell fate in parallel and independent routes. Interestingly, autophagic and apoptotic cell death pathway depends largely on the status of cell and the intracellular signaling environment, as wild type or drug resistant gastric cancer cells may decide to undergo apoptotic and autophagic cell death, respectively [59]. Second, autophagy may execute cell death with apoptosis in a cooperative manner [60]. In support of such hypothesis, Xu et al. showed that the anti-cancer drug akebia saponin PA resulted in both autophagic and apoptotic cell death in gastric cancer AGS cells [61]. Autophagy blockage suppressed the apoptosis-related caspase-3 activation, while inhibition of caspase-3 had no influences on autophagic pathway [61]. Relevantly, a recent report showed that disturbance of p62/SQSTM1, the autophagy adaptor, led to cargo loading failure, converted the cytoprotective clearance into insufficient autophagic degradation, and ultimately resulted in cell apoptosis [62]. Hence, it is possible that both autophagy and apoptosis modulate cell death independently, and autophagy lies on the upstream of apoptosis and is required for apoptotic cell death.

In the second scenario, autophagy acts as an oncogenetic pathway that favors tumor cell survival. The addition of autophagy inhibitor, such as chloroquine and bafilomycin A1, sensitizes the cytotoxicity of anti-cancer drugs in gastric cancer cells [63-68], indicating the autophagy inhibitor might be a favorable candidate for the chemotherapy of gastric cancer. The specific mechanism underlying the pro-survival function of autophagy remains largely unknown. One study has indicated that autophagy antagonizes apoptotic cell death in gastric cancer cells through mediating high-mobility group box-1 (HMGB1) release into the extracellular milieu [69]. Another issue important to consider is the chemoresistance in gastric cancer [70]. Recently, emerging lines of evidence highlight the involvement of autophagy in chemoresistance of gastric cancer. Apparent overactivation of autophagy is observed in chemoresistant gastric cancer cell line, whereas autophagy blockage by chloroquine significantly enhances the chemosensitivity of cells [71]. Consistent with this notion, sustained expression of Atg5 is detected in chemoresistant gastric cancer cell line, and downregulation of Atg5 sensitizes chemoresistant cells to drug therapy [72]. Given the fact that autophagy ensures the survival of gastric cancer cells that are resistant to chemotherapy, drug resistance, the major problem of chemotherapy associated with poor chemotherapy responses and prognosis, might be solved by autophagy inhibition [73].

Taken collectively, autophagy is a double-edged sword for gastric cancer. The pro-death (tumor suppressor) and pro-survival (tumor promoter) role of autophagy is particularly associated with its interaction of apoptosis. It is unclear, however, whether the indentified points of convergence between two pathways [74] also regulate these cell-death machineries in gastric cancer cells. Study about the impact of convergent points or molecular switch nodes, such as B-cell lymphoma-2 (Bcl-2) family members [75], in deciding the fate of gastric cancer cells, may provide new clue to unmask the crosstalk between autophagy and apoptosis in oncogenesis.

## AUTOPHAGY IN GASTRIC CANCER PROGRESSION

**Figure 2:**
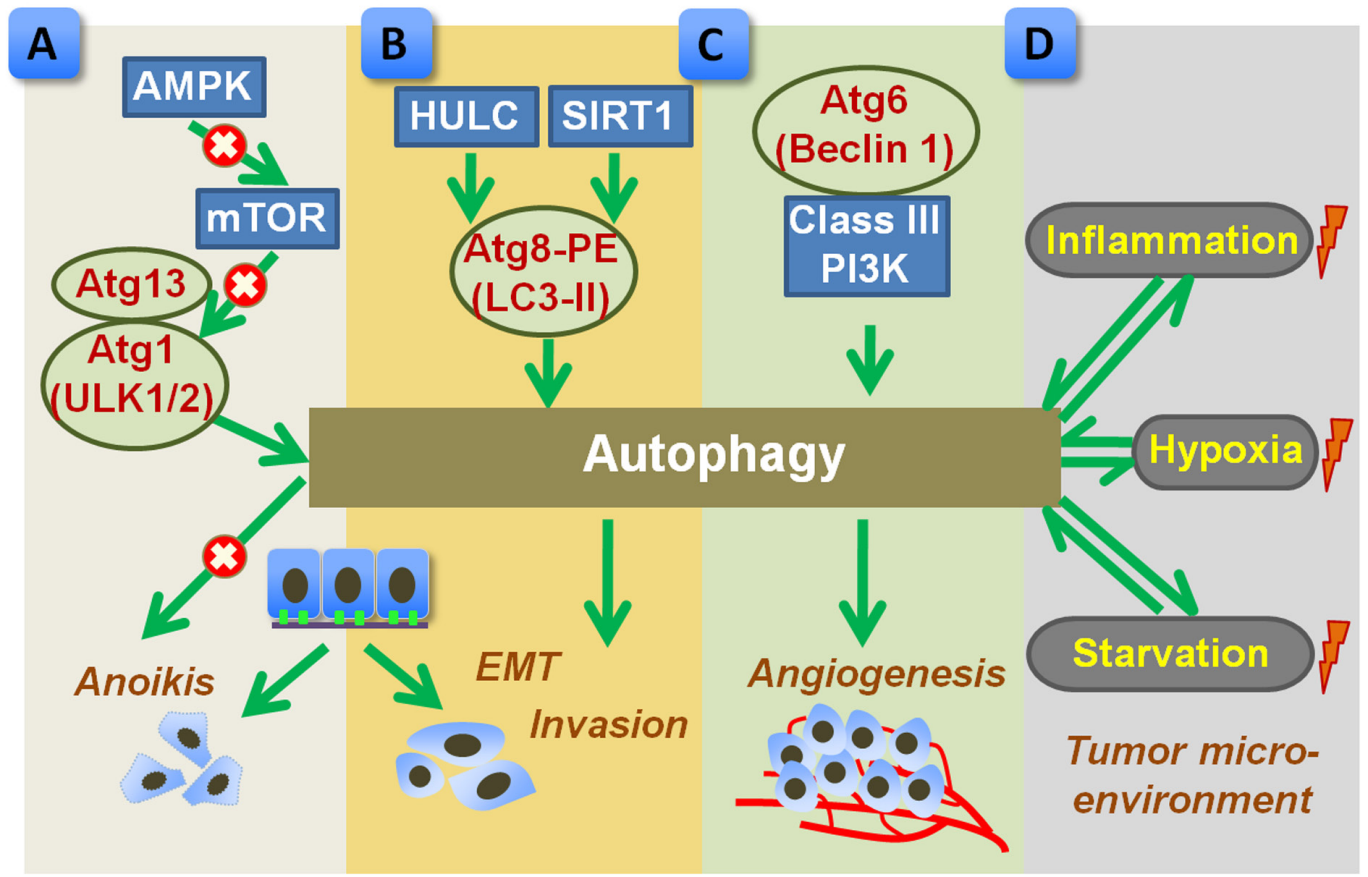
Proposed role of autophagy in promoting metastasis of gastric cancer **A.** Autophagy, regulated by AMPK-mTOR signal pathway, benefits the detached cancer cells to overcome anoikis. mTOR negatively regulates autophagy by inhibiting Atgs such as Atg13 and Atg1 (ULK1/2). **B.** Upstream factors, such as SIRT1 or HULC, trigger autophagic activation by inducing Atg8 and PE conjugation (LC3-II formation), which subsequently promotes EMT and tumor invasion. **C.** Autophagy, regulated by Atg6 (Beclin 1) and class III PI3K, enhances tumor angiogenesis. **D.** Tumor microenvironment, such as hypoxia, starvation, and inflammation, may influence autophagic process, which in turn shapes tumor microenvironment. The autophagy related proteins were highlighted in red. Arrow indicates a stimulatory effect and × indicates an inhibitive effect. AMPK, AMP-activated protein kinase; EMT, epithelial-to-mesenchymal transition; HULC, highly upregulated in liver cancer; LC3, microtubule-associated protein 1 light chain 3; mTOR, mammalian target of rapamycin; PE, phosphatidylethanolamine; PI3K, phosphatidylinositol 3-kinase; SIRT1, silent mating type information regulation 1.

Tumor metastasis, at the site of distant tissues or organs, is a sign predicting advanced progression and poor prognosis of gastric cancer [76]. The process of tumor metastasis is complex, involving a series of pathological events such as the breakdown of extracellular matrix (ECM), epithelial-to-mesenchymal transition (EMT), tumor angiogenesis, tumor microenvironment formation, etc [77]. The role of autophagy in tumor metastasis is believed to be both pro-metastatic and anti-metastatic [77]. Regarding gastric cancer, although autophagic cell death may inhibit metastasis, most of the current findings supports the notion that autophagy facilitate tumor metastasis through affecting several aspects (Figure 2).

### ECM degradation

Matrix metalloproteinase (MMP) proteins gain widespread attention because of their principle roles in the modulation of ECM degradation, paving the way for tumor cell metastasis [78]. In disseminated gastric cancer NUGC4 cells, treatment of chemokine CXCL12 resulted in an increased production of MMP and augmented cell migration through activating the mammalian target of rapamycin (mTOR) pathway [79]. It should be noted that mTOR is considered as a negative regulator of autophagic machinery. Under nutrient-rich condition, mTOR suppresses autophagy by inhibiting Atg13 and Atg1 (ULK1/2) complex, while blockage of mTOR upon nutrient starvation or stress launches autophagy in eukaryotes [80]. According to Hashimoto's report [79], inhibition of mTOR signal by rapamycin prevented the MMP generation, inhibited cell migration, and led to autophagic cell death in NUGC4 cells. In addition to pharmacological interference, enforced mTOR inhibition can be resulted from several upstream signals, such as the cellular energy sensor AMPK [81, 82]. Blockage of mTOR by AMPK triggers autophagy, which acts a pro-survival role for cancer cells during ECM detachment [83, 84]. The enhanced autophagic process prevents ECM detached cells from anoikis and contributes to luminal filling possibly by providing sustained ATP source [83, 84]. Hence, in the early stage of progression, autophagy might be an adaptive strategy for the detached cancer cells to overcome anoikis [85] (Figure 2A).

### EMT

Highly upregulated in liver cancer (HULC), a long non-coding RNA (lncRNA), is found to be overexpressed in gastric cancer cell lines as well as in gastric cancer tissues when compared to control [86]. Moreover, the increased HULC expression contributes to enhanced EMT phenotype and invasion in SGC-7901 cells by inducing LC3-II formation and autophagy induction [86], implying the involvement of autophagy in tumor metastasis. Similar to that of HULC, silent mating type information regulation 1 (SIRT1), a class III histone deacetylase, is also upregulated in tumor tissues and correlates with advanced lymph node metastasis in gastric cancer [87]. *In vitro* study further confirmed the regulatory effects of SIRT1 in EMT and invasion capability of gastric cancer cells [88]. Importantly, SIRT1 is a well characterized autophagy mediator, which drives the initiation of autophagy *via* deacetylation of Atgs, such as LC3 [89]. Based on the evidence, autophagic process, modulated by SIRT1 or other mediators may play a central role in tumor progression by regulating EMT and tumor cell invasion [90] (Figure 2B). Perturbation of autophagic activity may be a potential strategy for controlling tumor invasion.

### Tumor angiogenesis

Vasculogenic mimicry, a novel pattern of tumor angiogenesis, plays an essential role in the growth and metastasis of cancer by providing oxygen and nutrient supplementation [91]. Using a three-dimensional culture system in which gastric cancer SGC-7901 cells were maintained on Marigel matrix, Ding et al. [92] reported that autophagy promoted the survival and invasive ability of SGC-7901 cells by facilitating the formation of vasculogenic mimicry. Inhibition of autophagy by silencing of Beclin 1 expression reduced the cancer cell survival and invasion [92]. Hence, Beclin 1-dependent autophagy might be required for tumor angiogenesis of gastric cancer (Figure 2C). As tumor angiogenesis is in high nutrient and energy demands, autophagy supports bioenergetic demands by digesting and recycling of intracellular materials. Pharmacological inhibition of autophagy together with anti-angiogenic therapy merges as a promising way for overcoming tumor angiogenesis.

### Tumor microenvironment

Autophagy is an adaptive response to stress, and can be induced by a variety of stimuli. Regarding the tumor microenvironment, autophagy is a consequence rather than cause, that can be robustly induced by hypoxia, nutrient depletion, and inflammation [5]. Activated autophagy, in turn, shapes the tumor microenvironment by promoting tumor angiogenesis, providing nutrient supply, and regulating inflammatory responses [4, 5] (Figure 2D). Nonetheless, evidence regarding the involvement of autophagy during the formation of tumor microenvironment in gastric cancer is quite limited, and the precise role of autophagy in shaping tumor microenvironment still needs to be further clarified.

### Prognostic value of autophagy-related proteins in gastric cancer

Several molecular markers have been identified to possess prognostic value for gastric cancer, including MSI, growth factors (e.g. human epidermal growth factor receptor-2 [HER-2], epidermal growth factor receptor [EGFR], vascular endothelial growth factor [VEGF], etc.), cytokines (e.g. interleukin [IL]-11, IL-6), cell cycle mediators (e.g. cyclin E), apoptosis-associated regulators (e.g. Bcl-2, Fas, survivin), microRNAs (e.g. microRNA [miR]-21, microRNA-214, microRNA-433, etc.) [93]. Of greater concern is the accuracy and specificity of these biomarkers for delineating disease prognosis. Here, we mainly discuss the prognostic impact of three principle modulators in autophagy process.

Beclin 1 functions as a scaffold for the formation of phosphatidylinositol 3 kinase (PI3K) complex, which is an initial step for autophagosome formation. Binding of Bcl-2 family members (e.g. Bcl-2 and Bcl-xL) to Beclin 1 suppresses the induction of Beclin 1-dependent autophagy [94]. A Chinese study indicates that the level of Beclin 1 is closely correlated to the clinicopathologic characteristics of gastric cancer, including gender, age, and the histological subtype of tumor [41]. However, another Korean study shows that the Beclin 1 level is not correlated with the clinicopathologic properties of patients with gastric cancer, including metastasis, invasion, and stage [38]. Such discrepancy might be attributed to the geographical diversities or the small sample size studied. It may be fruitful to adopt larger sample size in future study. Beclin 1 is identified as an independent prognostic factor for gastric cancer [41, 95, 96]. Beclin 1 expression is negatively correlated with the poor tumor differentiation, tumor metastasis, advanced tumor-node-metastasis (TNM) stage, tumor recurrence as well as shorter survival in gastric cancer patients [41, 96, 97]. Consistently, decreased Beclin 1 level, closely associated with upregulated Bcl-xL expression, predicts increased malignant phenotype and poor prognosis of gastric cancer [95, 97]. In the patients with node-positive gastric cancer, Beclin 1 level is significantly associated with intravascular embolus and positively correlated to longer survival [42]. Based on these observations, increased Beclin 1 expression predicts a more favorable prognosis. Indeed, autophagy allows prolonged survival [98], making it amenable to explaining patient with reduced Beclin 1 expression have poor prognosis.

Increased number of LC3-positive puncta can be found in gastric cancer cells. In contrast to Beclin 1 that predicts better prognosis, in gastrointestinal cancers, upregulated LC3 expression partially correlates with the level of Ki-67, a proliferation index associated with the clinical courses of cancer [99]. The number of LC3-positive puncta is positively associated with the risks of tumor relapse after radical resection in patients with stage I-III gastric cancer, and negatively correlated to the overall survival rate for patients at stage IV [100]. Thus, the presence of LC3-positive puncta is proposed to be another independent biomarker predicting poor prognosis of gastric cancer [100]. The observation of accumulated LC3-positive puncta may be better explained in terms of impairments in autophagic degradation rather than enhancement of autophagic activity.

p62/SQSTM1, an ubiquitin binding protein, serves as a selective autophagy substrate [101]. The degradation of p62/SQSTM1 reflects the turnover of autophagy machinery, and defective autophagy results in abundant p62/SQSTM1 accumulation. The expressions of both p62/SQSTM1 and ubiquitin are very weak in normal gastric tissue samples, while increased nuclear and cytoplasmic expression of both proteins are detected in gastric cancers [102]. Moreover, the increased expression of p62/SQSTM1 is found to be associated with poor differentiation and reduced lymph node metastasis of gastric cancer [102]. This pattern of results concur with the notion that autophagy defect results in the insufficient degradation of p62/SQSTM1, which ultimately leads to tumorigenesis by dysregulating NF-κB signaling transduction pathway and gene expression modulation [33].

## CONCLUSIONS

Despite the studies on autophagy and gastric cancer have merged in the recent years, we are still at the very initial stages of understanding the complicated regulatory role of autophagy in the oncogenesis, progression and prognosis of this malignancy. Apoptosis is not the sole route of programmed cell death (PCD), but interacts with autophagy to execute gastric cancer cell death. In this regard, manipulation of autophagy has been proven to sensitize cancer cells to chemotherapy. However, autophagy can be more beneficial, rather than destructive, to enable cancer cell survival by providing energy. Given the dual role of autophagy in gastric cancer, it seems that the activation of autophagy should be maintained at a moderate degree. Nowadays, the development of molecules and pharmacologic agents targeting autophagy attracts increasing attentions and the autophagy inducers as well as inhibitors are currently under development for clinical use [103-105]. Interference of autophagic process may offer a novel strategy for controlling tumorigenesis and progression of gastric cancer.
